# Supplementary material for: Chromothripsis during telomere crisis is independent of NHEJ, and consistent with a replicative origin
Source: Genome Res. 2019 May;29(5):737–49. doi: 10.1101/gr.240705.118 (PMC6499312; doi:10.1101/gr.240705.118)
Supplement: Supplemental Material [file supp_gr.240705.118_Supplemental_file_1.zip › contigs/annotated_contigs/DB55/contig.2.DB55_length_285_mean_cov_5.26315789474.docx]

**DB55_length_285_mean_cov_5.26315789474**

```
TAAGGCATATATATATGCATATATATATATATATACTATATATATAAAATACAAAAACAAATGTTATATATATAACAATATAAAATACA
 >chr13:72127701-72127819 - E=9e-60
AAAATATAAAAATACAAAATGCAAAAACA|TTCCCTCATTCTAAATATAGTGGAATAGGCTGTAATAATTGACATTTGTGGTATATAAA
                               >chr13:72126652-72126819 - E=3e-83
TAGGCTCACTGTTTCTTGATGCTAGAGCTCTCTGTTTATCCTCCACACATTTGTCCACCGACGCTAGTTTACCGTGTTGCTCTCTCATG

CATAATTTGTTTTGGTTTT
```
